# Editorial: Recent advances in nanoradiopharmaceuticals for molecular imaging and radiotherapy

**DOI:** 10.3389/fmed.2024.1412600

**Published:** 2024-04-23

**Authors:** Nicolas Lepareur

**Affiliations:** ^1^Comprehensive Cancer Center Eugène Marquis, Pharmacy Department, Rennes, France; ^2^University of Rennes, Inrae, Inserm, Institut NUMECAN (Nutrition, Métabolismes et Cancer) – UMR_A 1341, UMR_S 1317, Rennes, France

**Keywords:** drug delivery, molecular imaging, multimodality, nanomedicine, nuclear medicine, radiolabeled nanomaterials, radionuclide therapy, radiopharmaceuticals

The combination of nanotechnology with nuclear medicine offers attractive diagnostic and therapeutic opportunities for the management of various conditions, notably cancer. Radiolabeled nanoparticles represent an innovative class of radiopharmaceutical agents holding great promises, by exploiting the intrinsic advantages of nanoparticles, such as increased surface area-to-volume ratio, targeting ability, high payload, and the potential for multiplexing several functionalities within one construct (multimodal imaging, combination therapies, or a mixture of both for theranostics), to provide groundbreaking medical solutions to address unmet medical needs.

This Research Topic for Frontiers in Medicine seeks to provide an overview of recent progresses in the field of nanomedicine and radiopharmaceuticals, with new approaches to the design and formulation of innovative radiolabeled nanoconstructs and their applications, advantages, limitations and future prospects, and challenges. Research on the combination of different radionuclides or radiopharmaceuticals with passively or actively targeted nanoparticles offers the possibility to increase personalized diagnostic efficacy and radiotherapeutic index.

Rare earth elements, i.e., scandium, yttrium and the 15 lanthanoids, are a family of elements which has proven to be particularly useful for cancer management. Wang and Li performed an extensive analysis of the literature dealing with the application of rare earth elements in cancer diagnosis and treatment. It ranged from lanthanide-doped nanomaterials to therapeutic radiopharmaceuticals, using especially yttrium-90, an electron-emitting nuclide particularly suited for radioembolization of liver cancers.

Another lanthanide that emerged as particularly attractive for cancer treatment is lutetium-177. Encapsulating both this nuclide and a drug, regorafenib, in PLGA nanoparticles, moreover decorated with receptor-specific ligands, targeting the cancer-overexpressed CXCR4 chemokine receptor, Cruz-Nova et al. demonstrated the interest of the combination of chemotherapy and radiopharmaceutical therapy in a colorectal cancer model.

^99m^Tc-sulfur colloids are a routinely used type of radiolabeled nanoparticles, valuable for a variety of indications, such as gastric emptying, sentinel lymph node scintigraphy, or assessment of functional liver reserve. Klinkert et al. presented us a case report of a patient initially diagnosed with a pancreatic tumor. Using both this agent and a radiolabel somatostatin analog, the authors could conclude it was in fact an intrapancreatic accessory spleen, thus highlighting the clinical usefulness of this diagnostic radiotracer.

Finally, a mini-review by Dixit et al. reported the different types of nanoconstructs and targeting strategies currently employed for nanoradiopharmaceuticals, to take advantage of both medical technologies, emphasizing the diversity of investigated approaches.

## Author contributions

NL: Conceptualization, Data curation, Formal analysis, Funding acquisition, Investigation, Methodology, Project administration, Resources, Software, Supervision, Validation, Visualization, Writing – original draft, Writing – review & editing.

